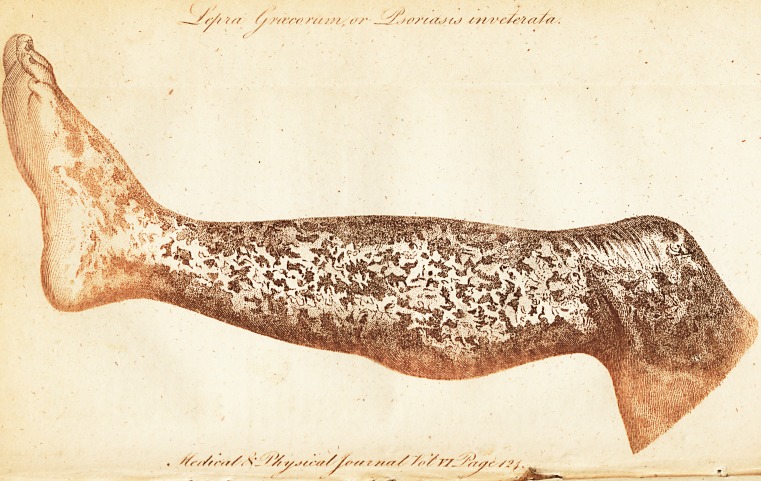# A Case of Lepra Grecorum, or Psoriasis Inveterata

**Published:** 1801-08

**Authors:** Charles Pears


### *A Case of Lepra Grecorum, or Psoriasis Inveterata;* by Charles Pears, F. M. S. &c. &c.

[ With an Engraving. ]

---

To the Editors of the Medical and Physical Journal.

Gentlemen,

HAVING met with a severe case of that singular disease, called the *Lepra Grecorum*, I was anxious to preserve the appearances

*Lepra Græcorum, or Psoriasis inveterata.*

appearances of it, and therefore obtained the drawing which accompanies this communication. Your obliging readineſs in accepting of what I have hitherto ſent, induced the hope that this would not be refuſed; and ſhould it be ſo far approved as to be made the ſubject of an engraving for more accurate delineation, I ſhall eſteem myſelf highly honoured; and only beg that the following account of the caſe, (drawn up in ſo much haſte as to preclude the poſſibility of adding thoſe obſervations I meant to have ſent) will be received as a ſufficient explanation, and that I may be allowed the indulgence of another communication for the remainder, at a future opportunity.

In July 1800, I was applied to by Thomas Stubbs, aged 49, who thus deſcribed his ſituation. He was attacked ſix years ſince with a ſenſe of ſtiffneſs in the legs and arms. The cuticle deſquamated in pieces equal to the ſize of two fingers in breadth. This continued for ſome weeks, and occaſioned ſuch a degree of debility, as to confine him to his bed; after which he recovered, and continued well for the ſpace of four or ſix weeks. Another attack then commenced. The *nails* were ſhed, and ſucceeded by new ones. The patient *sometimes* experiences pain, but this has not been the caſe lately. His appetite is good. He is reſtleſs, from an *itching* which prevails all over the body. Theſe attacks generally recur every three months; and during their continuance, a quart of the cuticle has fallen from the body in branny flakes every night, for a fortnight or three weeks, and *half a pint* is removed from the bed *two or three times* during the day.

His bowels are regular. The debility is ſuch as to prevent his working as a carpenter, and "when he ſtands long at the bench," pain follows the exertion. In 1796-7, he was admitted a patient in St. Thomas's Hoſpital, but diſcharged withoutbeing relieved: and alſo from the Surry Diſpenſary, where he afterwards applied. His *hands* are ſometimes ſo bad, and the fiſſures in them ſo deep, that all motion is prevented. The cuticle on theſe parts has deſquamated in the form of a glove. From the feet alſo, in the ſhape of thoſe parts. *The whole* of the body ſuffers this deſquamation, *except the pubes*, *scrotum*, &c. where it never has taken place; but where it has lately began to ſhew a diſpoſition thereto. The face and head are affected as other parts of the body, more eſpecially the roots of the hair. The return of every attack is preceded by a violent itching, which always indicates its approach.

The general appearance of the body is of a dark or reddiſh brown, which appears more ſtrongly marked when expoſed by the the recently fallen off white and branny cuticle; evincing a more ſtriking contraſt.

For the removal of this ſevere diſeaſe, I gave the following:

℞. Hydrargyr. muriati. gr. x. Spt. ætheris nitros. ℥j. Tinct. opii. [illegible]j. M. Cap. gtt. x. bis in die. ℞. Hydrargyr. muriat. gr. iij. Aq. fontan. lb. ij. M. ft. lotio. To be applied every night to the parts affected.

By Sept. the 8th. the ſkin became much ſmoother, the ſcales or flakes of deſquamating cuticle were much leſs, and the patient generally much better, being able to walk, and alſo to work in a degree. The ſenſible heat of the ſkin was much leſs.

Oct. 13th. He was ſeverely attacked with cholera morbus, on which account I diſcontinued the hydrar. mur. but the uſe of the *catechu* having recovered him by the 15th inſtant, he again reſumed the uſe of the former medicine.

24th. The red ſpots, inſtead of enlarging and producing *scales*, died away, and left the ſkin ſmooth and clear.

Dec. 20th. The patient diſbelieving that the medicine could produce ſuch a *speedy effect*, I ordered its uſe to be *discontinued*, in order to evince its power over the diſeaſe, which returned ſo ſoon with aggravated violence, that he immediately acknowledged his miſtake, and begged for his medicine. Since this time he has continued the uſe of his mixture and lotion, and found the attacks of the diſeaſe recur leſs frequently, and at longer intervals. The laſt, which occurred in March laſt, continued about four weeks, ſince which time he is again recovered, and finds himſelf in good health, continues his work as uſual, and his body is in a very good and favourable condition. The doſe has been gradually increaſed to 15 drops, three times a day.

So far does the hiſtory of this caſe reach the preſent time. Its future procedure muſt conſequently be an after recital, which I ſhall the more anxiouſly await, from the opinion of ſome, that *mercury is always ineffectual, and generally hurtful*, however contradicted by the ſucceſsful practice of others: ſo that the diſtinction thus derived, of its difference from *syphilis*, is not well founded.

*Thomas* * ſays, "This truly horrid and loathſome diſorder, which ſeems peculiar to warm climates, is evidently of a very *contagious* nature." But this does not ſeem to be the caſe here, in colder climates; for the numerous family of the above patient,

* See Thomas's *Medical Advice to the Inhabitants of warm Climates*, p. 119.

patient, uſing every domeſtic utenſil after him, are never affected by it. *Lommius** obſerves, that "the fourth ſpecies of *impetigo*, called the leproſy, is of all others the moſt terrible, becauſe it is *incurable.*" This alſo appears to be, happily, an untruth, as the above inſtance added to others may evince. Omiting, however, to enlarge further at this time, I ſhall only obſerve, that the drawing is ſo far from being an *aggravated* repreſentation of the limb, which *fairly* reſembled the whole body, that it is not nearly ſo bad as the parts really were, both before and afterwards.

*Rockingham Row, Newington Butts,*

*April,* 1801.

I remain, &c.
C. PEARS.

* Vide Jodocus Lommius's *Medicinal Obſervations,* Winter's Tranſlation, Book 2. p. 184.

## Figures and Tables

**Figure f1:**